# William Robert Ackland

**Published:** 1949-07

**Authors:** 


### Obituary

### WILLIAM ROBERT ACKLAND, M.D.S. (BRISTOL), M.R.C.S.

WILLIAM ROBERT ACKLAND died on July 26th at the age of eighty-five. He was educated at Torquay, at Charing Cross Hospital and at the Royal Dental Hospital, where he won the Saunders Scholarship and many prizes. He took the L.D.S. in 1884 and the M.R.C.S. in 1886, and was soon after appointed Assistant Dental Surgeon to Charing Cross Hospital. In 1888 he was elected the first Honorary Dental Surgeon to the Bristol Royal Infirmary, settled in Bristol and joined the Bristol Medico-Chirurgical Society. He was a member also of the Odontological Society, later the Odontological Section of the Royal Society of Medicine (President, 1922) and the British Medical Association (President of the Bath and Bristol Branch, 1914 and again 1933). He made many contributions to discussions and to professional journals, especially on the relationship between dental and general disease : and devised a set of instruments for the drainage of the maxillary antrum through a tooth-socket. During the 1914 war he was appointed Major, R.A.M.C., on the staff of the Second Southern General Hospital. He was a member of the Dental Board and also of the General Medical Council, a very active member of the teaching staff of Bristol University and promoter of the University Dental Hospital. In 1913 he was awarded the degree of M.D.S., Bristol : and in 1924 was appointed to the Consulting Staff of the Bristol Royal Infirmary.

Ackland added to a wide knowledge of his speciality an unusual charm of manner. In his younger days he was a keen fox-hunter : and always a lover of pictures and a generous patron of artists, as the walls of his house bore witness. Another feature of his home was a ball-room in which he gave memorable children's parties. He married in 1917 but his wife predeceased him.

